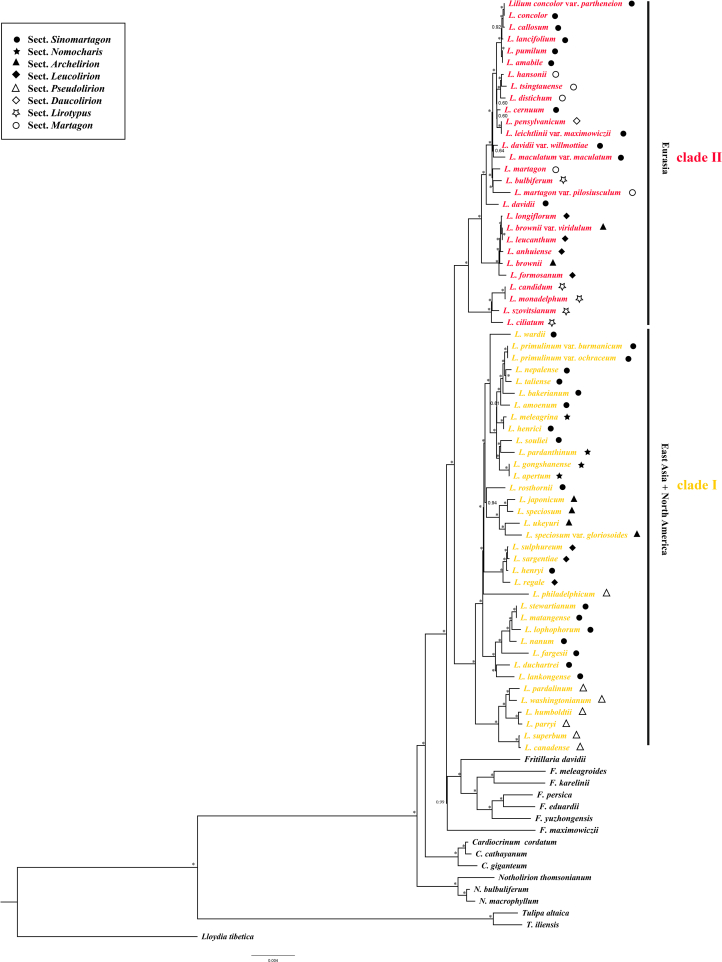# Historical biogeography and evolutionary diversification of *Lilium* (Liliaceae): New insights from plastome phylogenomics

**DOI:** 10.1016/j.pld.2023.07.009

**Published:** 2023-08-03

**Authors:** Nian Zhou, Ke Miao, Changkun Liu, Linbo Jia, Jinjin Hu, Yongjiang Huang, Yunheng Ji

**Affiliations:** a CAS Key Laboratory for Plant Diversity and Biogeography of East Asia, Kunming Institute of Botany, Chinese Academy of Sciences, Kunming, Yunnan 650201, China; b University of Chinese Academy of Sciences, Beijing 100049, China; c Key Laboratory of Bio-Resources and Eco-Environment of Ministry of Education, College of Life Sciences, Sichuan University, Chengdu, Sichuan 610065, China; d Yunnan Key Laboratory for Integrative Conservation of Plant Species with Extremely Small Population, Kunming Institute of Botany, Chinese Academy of Sciences, Kunming, Yunnan 650201, China

**Keywords:** Asian monsoon, Climatic changes, Distribution range, Evolutionary complexity, Radiative diversification, Species diversity, Qinghai-Tibet Plateau (QTP)

## Abstract

Here, we infer the historical biogeography and evolutionary diversification of the genus *Lilium*. For this purpose, we used the complete plastomes of 64 currently accepted species in the genus *Lilium* (14 plastomes were newly sequenced) to recover the phylogenetic backbone of the genus and a time-calibrated phylogenetic framework to estimate biogeographical history scenarios and evolutionary diversification rates of *Lilium*. Our results suggest that ancient climatic changes and geological tectonic activities jointly shaped the distribution range and drove evolutionary radiation of *Lilium*, including the Middle Miocene Climate Optimum (MMCO), the late Miocene global cooling, as well as the successive uplift of the Qinghai-Tibet Plateau (QTP) and the strengthening of the monsoon climate in East Asia during the late Miocene and the Pliocene. This case study suggests that the unique geological and climatic events in the Neogene of East Asia, in particular the uplift of QTP and the enhancement of monsoonal climate, may have played an essential role in formation of uneven distribution of plant diversity in the Northern Hemisphere.

## Highlights

• The phylogenetic backbone of *Lilium* was reconstructed based on a large plastome data set.
• This study provides new insights into the historical biogeography and evolutionary diversification of *Lilium.*

## 1 Introduction

Plant diversity in the Northern Hemisphere is extremely uneven, with much higher diversity in East Asia than in Europe and North America (Latham and Ricklefs, 1993; Qian, 2001, 2002; Adams, 2009). To date, the geological and climatic events responsible for this uneven distribution of plant diversity remain poorly elucidated (Xing and Ree, 2017). One model system for exploring the historical events that may have led to this uneven distribution of plant diversity is the genus *Lilium* L. *Lilium* is a large genus of the monocotyledonous family Liliaceae that includes approximately 115 species of bulbous herbaceous perennials (https://powo.science.kew.org/) distributed throughout the middle and high latitudes of the Northern Hemisphere (Liang and Tamura, 2000). Extant *Lilium* species are typically distributed in temperate regions of the Northern Hemisphere; however, there are far more *Lilium* species in East Asia than in Central Asia, Europe, and North America (Liang and Tamura, 2000). The diversity center of the genus is East Asia (Liang and Tamura, 2000), where nearly half of currently recognized *Lilium* species occur, mostly in Southwest China and the Qinghai-Tibet Plateau (QTP; Wu et al., 2006; Rong et al., 2011). Understanding the evolutionary and biogeographical history of *Lilium* may provide new insights into how climatic and geological events have shaped the uneven distribution of plant diversity in the Northern Hemisphere.

*Lilium* currently lacks a robust phylogenetic backbone, impeding our understanding of the classification, evolution, and historical biogeography of the genus. Phylogenetic analyses of *Lilium* have been limited by two factors. First, infra-generic classification of *Lilium* has long relied on floral morphology, resulting in constant changes in the subdivisions of the genus (Endlicher, 1836; Wilson, 1925; Comber, 1949; Baranova, 1988). Second, previous studies have suggested that some evolutionarily complex events may have occurred during the evolution of *Lillium*, e.g., inter-sectional/specific hybridization (Gao et al., 2013; Gong et al., 2017; Huang et al., 2018) and/or morphological convergence (Liu and Sun, 2019; Givnish et al., 2020). Consequently, the generally accepted classification of the genus (Comber, 1949), which divides *Lillium* into seven sections (i.e., sect. *Archelirion,* sect. *Daurolirion*, sect. *Leucolirion,* sect. *Liriotypus*, sect. *Martagon*, sect. *Pseudolirium*, and sect. *Sinomartagon*), has recently been shown to create non-monophyletic section-level taxonomic units (Nishikawa et al., 1999; Gao et al., 2013; Du et al., 2014; Gong et al., 2017; Huang et al., 2018; Kim and Kim, 2018; Li et al., 2022). In addition, *Nomocharis* Franch., which was once a segregated genus, has been reduced as a section-level taxonomic unit of *Lilium* (Gao et al., 2012; Gao and Gao, 2016), inferred from the evidence of molecular phylogenetics (Gao and Gao, 2016; Huang et al., 2018; Li et al., 2022). However, whether the sect. *Nomocharis sensu* (Gao et al., 2012; Gao and Gao, 2016) is monophyletic remains unresolved. To reconstruct robust phylogenies for such phylogenetically challenging plant lineages, taxon sampling should be extended and alternative data sets with more phylogenetically informative variations used (Rosenberg and Kumar, 2001; Rokas and Carroll, 2005; Whitfield and Lockhart, 2007; Philippe et al., 2011).

High-throughput sequencing technologies have been increasingly used to generate genome-scale data for phylogenetic studies. Phylogenetic analysis of sequence data generated by high-throughput sequencing, such as complete plastid genomes (plastome) and genome-wide nuclear sequences, which possess orders of magnitude more sequence variations than single or multiple sequence regions produced by Sanger sequencing, have exhibited greater potential for resolving challenging relationships in a wide spectrum of plant lineages (e.g., Rokas and Carroll, 2005; Jansen et al., 2007; Moore et al., 2007, 2010; Parks et al., 2009; Folk et al., 2016; Morales-Briones et al., 2018; McKain et al., 2018; Stull et al., 2020; Ji et al., 2021; Wen et al., 2021; Su et al., 2023; Xu et al., 2023). Theoretically, phylogenetic reconstruction based on the uniparentally inherited plastome DNA sequences recovers only the maternal (or in some cases the paternal) relationships of a certain plant lineage, compared with the relatively integrated evolutionary schemes recovered by analysis of the biparentally inherited nuclear genome-scale data. Even so, with the widespread application of plastomes in phylogenetic studies, numerous historically difficult issues in plant phylogenetics have been satisfactorily addressed (Jansen et al., 2007; Moore et al., 2007, 2010; Parks et al., 2009; Huang et al., 2016; Carlsen et al., 2018; Li et al., 2019; Yang et al., 2019; Ji et al., 2021, 2023), indicating that plastomes are as important as nuclear genome data sets and will continue to play an indispensable role in plant phylogenetics.

Several recent studies have attempted to use completed plastome DNA sequences to clarify phylogenetic ambiguities in *Lilium* (Du et al., 2017; Kim et al., 2017, 2019; Duan et al., 2022; Li et al., 2022). Although analysis of plastome data sets has largely improved phylogenetic resolution, previous studies have failed to recover a robust phylogenetic backbone of the genus due to small species-level sampling size. Here, we aim to infer the historical events that have shaped the uneven distribution of *Lilium*. For this purpose, we used plastome sequence data from 64 currently accepted *Lilium* taxa (including seven previously recognized sections plus the section *Nomocharis*) to recover a phylogenetic backbone of the genus. We then used a time-calibrated phylogenetic framework to infer the biogeographical history and evolutionary diversification of the genus.

## 2 Materials and methods

### 2.1 Plant samples, DNA extraction and illumina sequencing

In total 64 currently accepted *Lilium* species were included in this study. The taxonomic sampling covers 55.65% extant species and the entire distribution range of the genus. Plastomes representing 14 species were newly sequenced (Table S1), the remaining were obtained from the publicly available GenBank database (last accessed on April 1st, 2023; Table S2). To avoid misidentification of species resulting in the bias of results, only sequences with available vouchers were selected. The original sources of the plant materials used in this study and their voucher information are presented in Table S1.

Total genomic DNA of plant samples were extracted from approximately 10 mg silica gel-dried leaves using the CTAB method (Doyle and Doyle, 1987). Paired-end libraries with an average insert size of approximately 400 bp were prepared using a TreSeq DNA Sample Prep Kit (Illumina, Inc., USA) according to the manufacturer's protocol. Shotgun sequencing was performed on the Illumina Novaseq 6000 platform to generate approximately 4 Gb of raw reads for each sample.

### 2.2 Plastome assembly and annotation

The software Trimmomatic v.0.40 (Bolger et al., 2014) was used to remove adapters and to filter low-quality Illumina reads using pre-set parameters. Clean reads were assembled into plastomes with the pipeline GetOrganelle v.1.7.7.0 (Jin et al., 2020), using the complete plastome DNA sequence of *Lilium taliense* (GenBank Accession Number: KY009938) as a reference. Assembly graphs were visualized and edited using Bandage v.0.8.0 (Wick et al., 2015). Assembled plastomes were annotated with GeSeq (Tillich et al., 2017). Positions for start and stop codons and the exon/intron boundaries were checked manually using Geneious v.10.2 (Kearse et al., 2012). Annotated tRNA genes were further verified with tRNAScan-SE v.2.0 (Chan et al., 2021). The multiple genome alignment program Mauve v.4.0 (Darling et al., 2004) was used to detect structural rearrangements among *Lilium* plastomes.

### 2.3 Phylogenetic analysis

The phylogenetic framework of *Lilium* was reconstructed based on 64 complete plastomes. The complete plastomes of 16 species (i.e., *Fritillaria*, *Cardiocrinum*, *Notholirion* and Tulipeae) were designated as outgroups (Do et al., 2020; Huang et al., 2018; Li et al., 2022). Complete plastome DNA sequences (including LSC, SSC, and two copies of IRs) were aligned using the MAFFT program (Katoh and Standley, 2013). Phylogenetic analyses were performed with maximum likelihood (ML) and Bayesian inference (BI) methods. ML phylogeny was reconstructed using IQ-Tree v.2.1.3 (Chernomor et al., 2016; Nguyen et al., 2015) under the TVM + F + R6 model, with 1000 rapid-search replicates to estimate bootstrap (BS) support for each node. BI analysis was performed using MrBayes v.3.22 (Huelsenbeck and Ronquist, 2001) and the TVM + I + G model recommended by ModelTest v.3.7 (Posada and Crandall, 1998) with the Akaike information criterion (Posada and Buckley, 2004). The BI analysis ran the Markov chain Monte Carlo (MCMC) algorithm for two million generations, and trees were sampled once every 1000 generations with the first 25% discarded as burn-in and the effective sample size (ESS) > 200. The posterior probability values (PP) were calculated based on the remaining trees. The resulting ML and BI trees were edited using FigTree v.1.4.3 (Rambaut, 2016).

### 2.4 Molecular dating and diversification rate estimation

Divergence times were estimated using BEAST v.1.10.4 (Suchard et al., 2018). Given the absence of well-documented fossils in Liliales, three calibration points referring to the results of Givnish et al. (2020) and Li et al. (2022) were used to calibrate the phylogenetic trees: (1) 25.16 million years ago (Ma) for the crown node of Liliaceae tribe Lilieae, (2) 22.89 Ma for the stem age of the *Lilium* + *Fritillaria* clade, and (3) 18.6 Ma for the stem age of *Lilium*. BEAUti v.1.10.4 (Drummond et al., 2018) was used to set criteria for molecular dating. The ML tree was fixed as the topological constraint in the BEAST analysis, using Yule process prior with the uncorrected lognormal relaxed clock model under GTR nucleotide substitution model. The MCMC simulations were run for 400 million generations with sampling every 1000 generations. The ESS was monitored by Tracer v.1.7.1 (Rambaut et al., 2018). After removing the first 20% as burn-in, independent trees were combined using the TreeAnnotator v.1.10.4 (Rambaut and Drummond, 2018).

The rate change of species diversification over time was inferred using the lineage through time (LTT) plot method, a visual tool to access patterns of diversity in time scales. The consensus chronogram inferred from the results of molecular dating was computed by APE v.5.6–2 package (Paradis and Schliep, 2019) in an R environment. Additionally, we quantified shifts in net diversification rate in *Lilium* by Bayesian Analysis of Macroevolutionary Mixtures (BAMM), which is entirely oriented towards detecting and quantifying heterogeneity in evolutionary rates (Rabosky et al., 2014). The complete plastomes of 16 species (i.e., *Fritillaria*, *Cardiocrinum*, *Notholirion* and Tulipeae) were designated as outgroups (Do et al., 2020; Huang et al., 2018; Li et al., 2022). Based on the time calibrated trees from BEAST, the BAMM analysis was conducted with BAMMtools v.2.1.9 (Rabosky et al., 2014).

### 2.5 Ancestral range reconstruction

The following five regions were defined for biogeographic analyses based on the distribution of extant *Lilium* species (A) Southwest China and QTP; (B) East, Central, South China and northern Indochina; (C) North China and Northeast Asia; (D) Central Asia and Europe; and (E) North America. The details are provided in Table S3. The ancestral range reconstruction was conducted using the S-DIVA method as implemented in the RASP v.4 software (Yu et al., 2020). The tree data from BEAST analysis were used as the input trees, and the maximum number of areas at each node was set to five. To avoid biased inferences caused by uncertainty in the root areas of the outgroups, outgroups were removed in advance using APE v.5.6–2 package (Paradis and Schliep, 2019).

## 3 Results

### 3.1 Phylogenetic relationships

Plastomes of *Lilium* species sampled in this study varied from 151,655 bp (*Lilium bakerianum*) to 153,235 bp (*Lilium fargesii*), with conserved gene content and genome rearrangement. These plastomes contained 114 unique genes, including 80 protein-coding genes, 4 rRNA genes, and 30 tRNA genes. *Lilium* plastomes showed consistent gene order and no structural rearrangements were detected (Fig. S1). ML and BI analyses based on complete plastome DNA sequences generated almost identical tree topologies with full support (BS = 100%; PP = 1.00) for the majority nodes (Fig. 1 and S2). Both ML and BI phylogenies recovered two major clades (BS = 100%; PP = 1.00), consisting of East Asian + North American species (Clade I) and Eurasian species (Clade II). Notably, neither the seven sections recognized by Comber (1949) nor the section *Nomocharis* *sensu* Gao and Gao (2016) was recovered as monophyletic by ML and BI phylogenies.

**Fig. 1:**
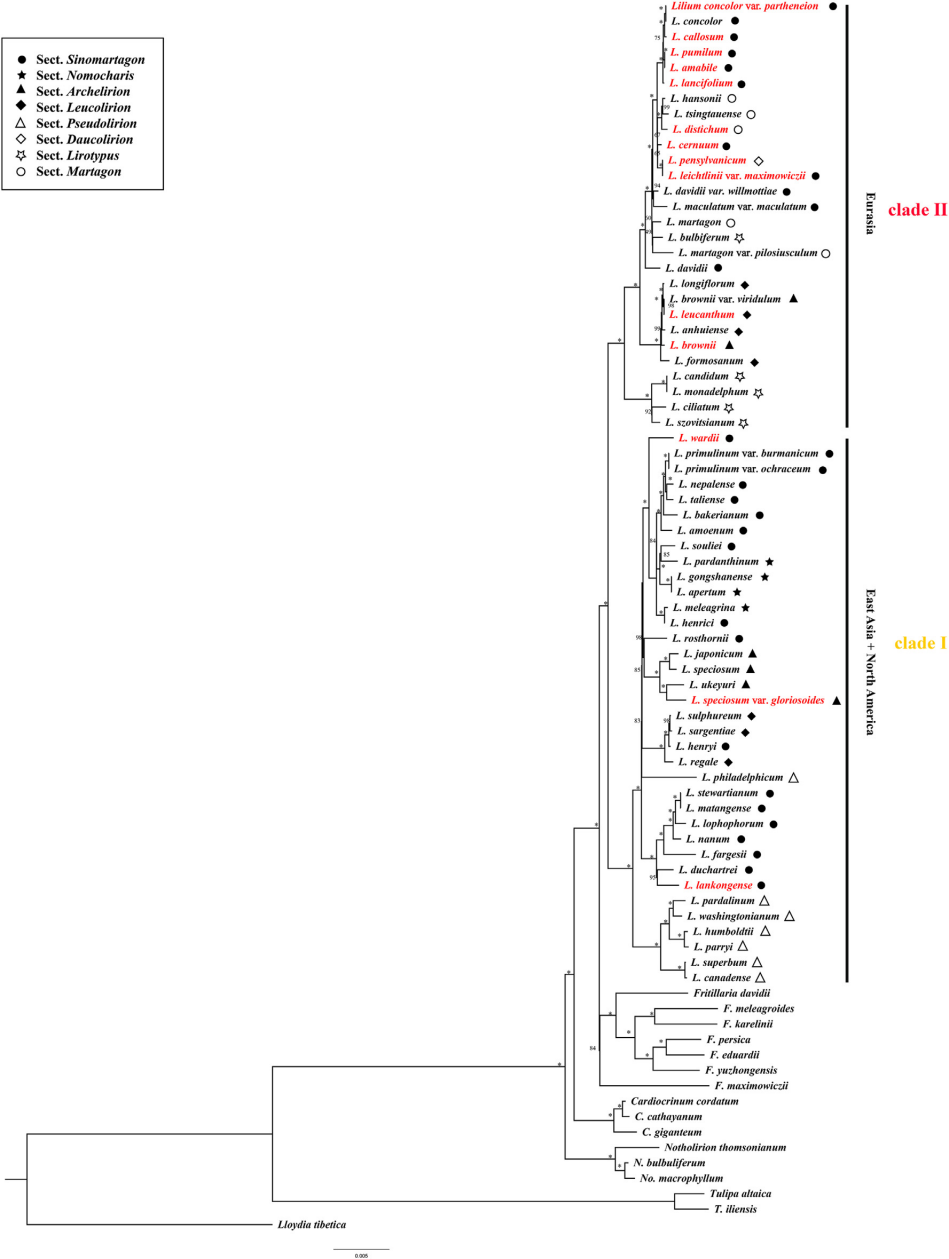
Maximum likelihood (ML) phylogeny of *Lilium* inferred from complete plastome DNA sequences. Numbers at nodes represent maximum likelihood bootstrap (BS) percentages. An asterisk indicates that BP is 100. The sectional treatments of Comber (1949) and the section *Nomocharis* defined by Gao et al. (2012) are indicated on the right. Species with newly sequenced plastomes are highlighted in red.

### 3.2 Molecular dating and historical diversification

**Fig. 2:**
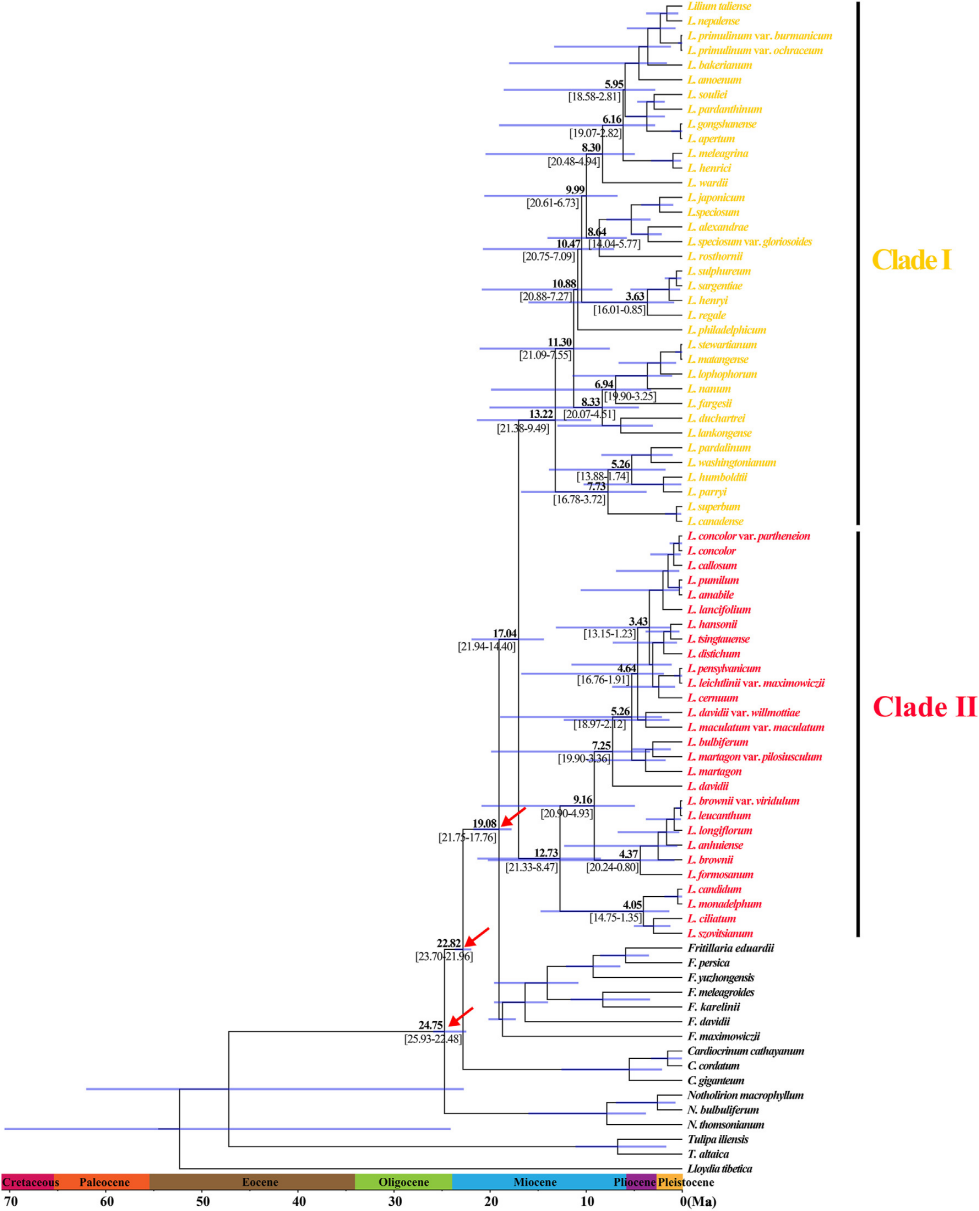
Molecular dating in *Lilium* based on complete plastome DNA sequences. Numbers above/under the tree branches represent mean divergent ages and 95% confidence interval of divergence times. Red arrows indicate the calibration points for the molecular dating (see text). Divergence time and the timeline are indicated in mega-annum (Ma).

**Fig. 3:**
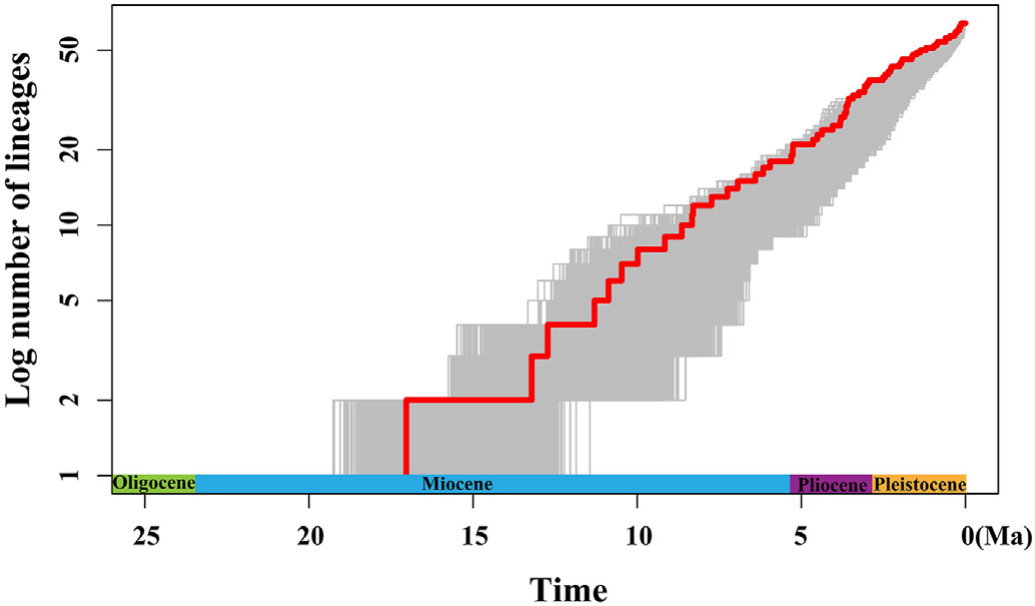
Lineage through time (LTT) plot analysis.

**Fig. 4:**
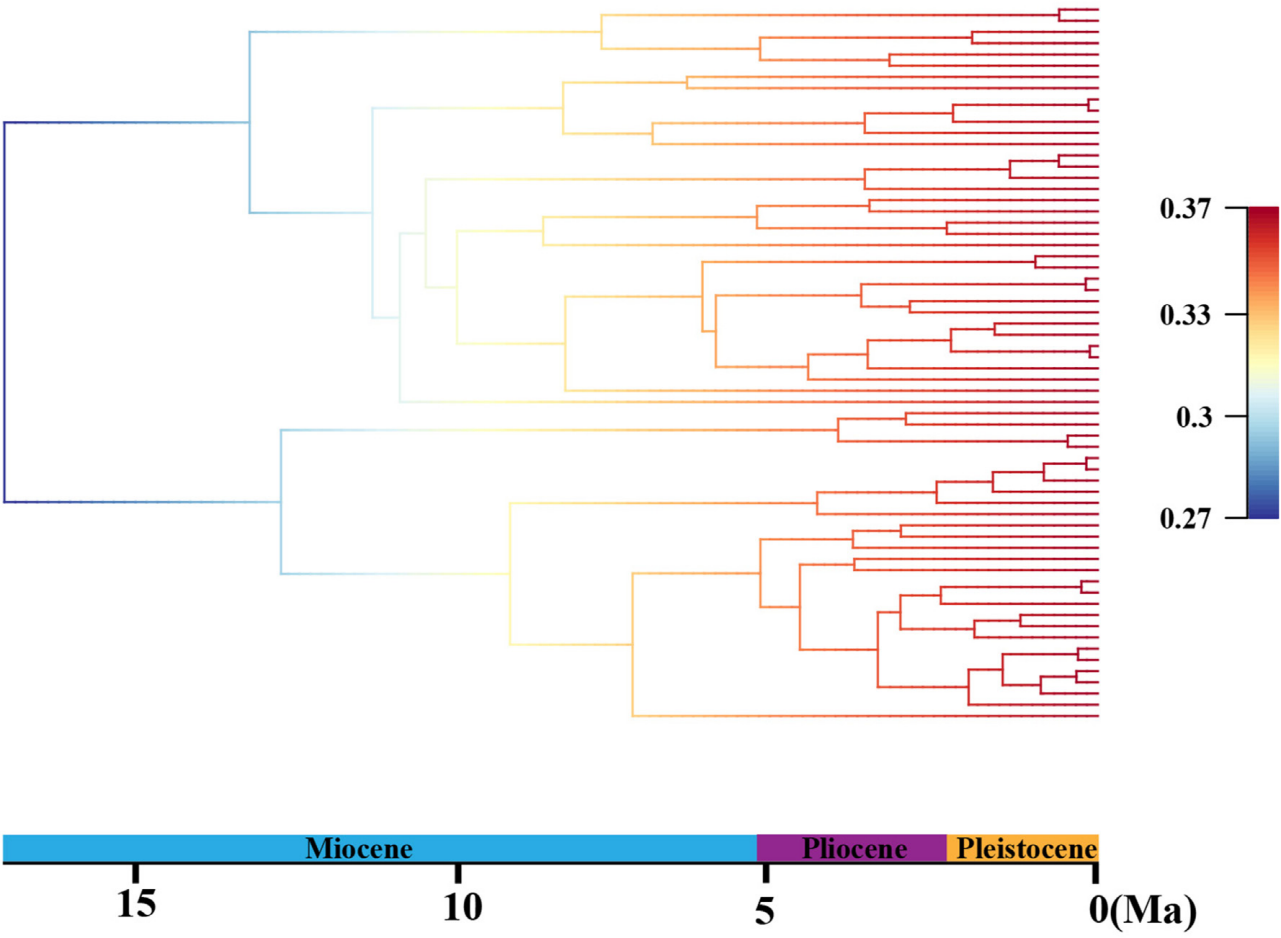
Bayesian Analysis of Macro-evolutionary Mixtures (BAMM) based on the time-calibrated maximum clade credibility tree from BEAST. Colors on the branch represent the mean of the posterior density of net diversification rate (speciation rate minus extinction rate).

BEAST analysis showed that the early divergence of *Lilium*, corresponding to the splitting of two major clades (Clade I and Clade II), occurred at 17.04 Ma (95% HPD: 21.94–14.40 Ma), around the middle Miocene (Fig. 2). The crown ages of Clade I and Clade II were dated at 13.22 Ma (95% HPD: 21.38–9.49 Ma) and 12.73 Ma (95% HPD: 21.33–8.47 Ma), respectively. Within each clade, the divergence of shallow branches, which resulted in the formation of most extant species, occurred in the late Miocene, Pliocene, and Pleistocene. LTT plot analysis indicated that the diversification rate was relatively stable during the early evolution of *Lilium*, but accelerated in the late Miocene (*ca.* 10.0 Ma) and continued increasing during the Pliocene and Pleistocene (Fig. 3). Similarly, BAMM identified an acceleration in net species diversification rate in *Lilium* about 10.0 Ma, which continued during the Pliocene and Pleistocene (Fig. 4).

### 3.3 Ancestral area reconstruction

**Fig. 5:**
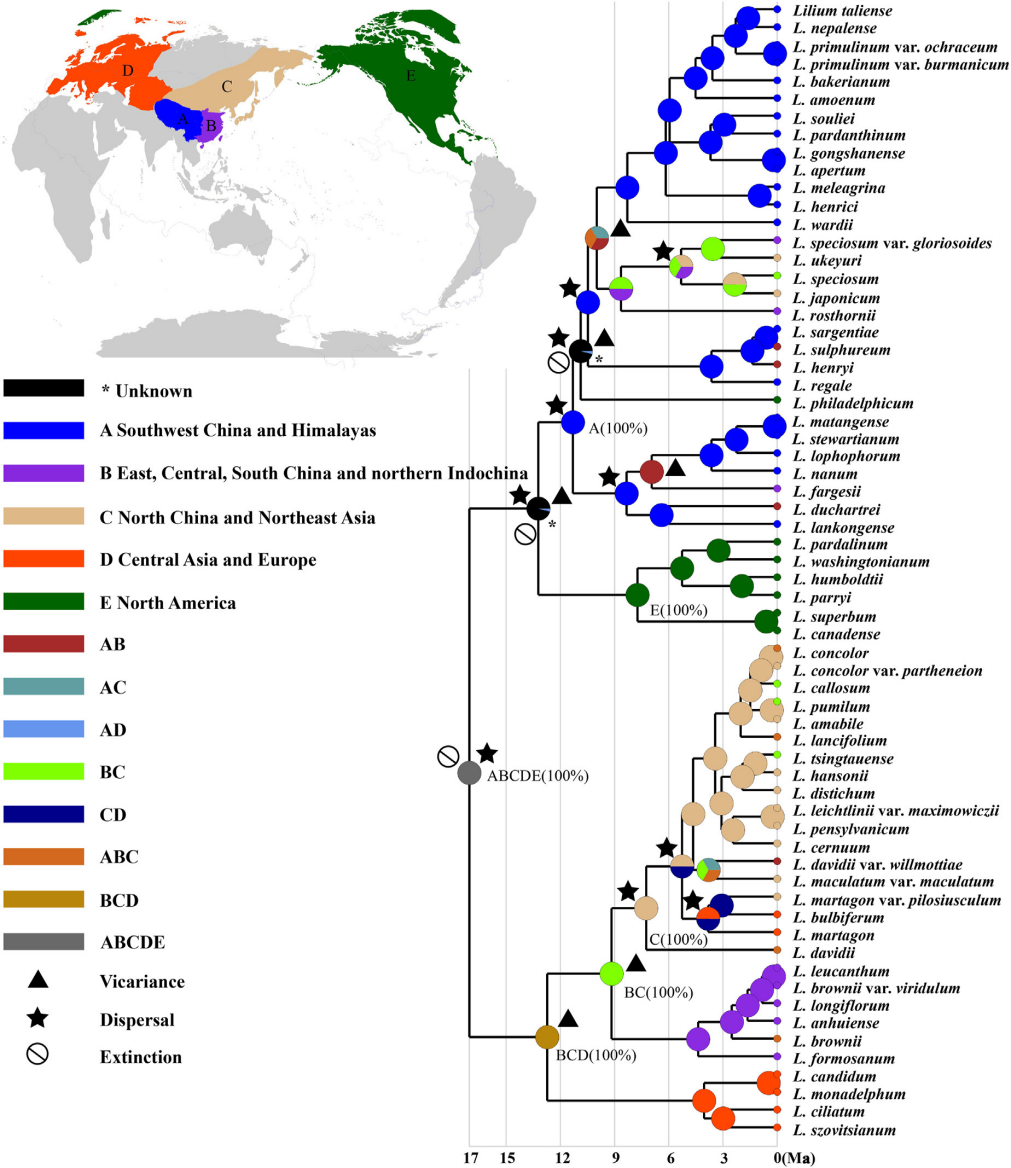
Reconstruction of ancestral area of *Lilium* using S-DIVA analysis inferred from plastid tree. *Lilium* species assigned to five areas based on their current distributions: A. Southwest China and QTP, B. East, Central, South China and northern Indochina, C. North China and Northeast Asia, D. Central Asia and Europe, E. North America.

Statistical-Dispersal Vicariance Analysis (S-DIVA) indicated that the most recent common ancestor of extant *Lilium* species sampled in this study occurred in Eurasia and North America (Fig. 5). Our analysis also indicated the evolution of the ancestral populations into clade I and clade II was mediated by distant dispersal followed by an extinction event. Although S-DIVA analysis failed to reconstruct the ancestral area of clade I, the preliminary formation of the disjunction distribution between North American and Southwest China and the QTP was inferred to be jointly driven by a dispersal, an extinction, and a vicariance event. We also inferred that the forces that drove the evolutionary changes in distribution of Clade I included five dispersal events, one extinction, and three further vicariances events. In addition, S-DIVA analysis showed that the ancestral populations of the Clade II mostly likely spread throughout Eurasia (from northern Indochina to Europe), and that their evolutionary trajectories were influenced by three vicariances and two dispersals.

## 4 Discussion

### 4.1 Non-monophyly of section-level taxonomic units within *Lilium*

In this study, well-supported and highly resolved intrageneric relationships within *Lilium* were recovered, with most nodes being robustly supported, providing a backbone phylogeny for critically exploring the classification of the genus. Similar to previous molecular phylogenetic investigations of *Lilium* (Gao et al., 2013; Du et al., 2014; Gong et al., 2017; Huang et al., 2018; Kim and Kim, 2018; Nishikawa et al., 1999; Duan et al., 2022; Li et al., 2022), this study failed to resolve the seven sections recognized by Comber (1949) and sect. *Nomocharis* defined by Gao and Gao (2016) as monophyletic. Consistent with previous studies (Gao et al., 2013; Gong et al., 2017; Huang et al., 2018), we found no significant conflicts between nuclear and plastid phylogenies (cytonuclear discordance). Researchers have proposed that the polyphyly of these section-level taxonomic units within the genus were likely caused by inter-sectional hybridization rather than by incomplete lineage sorting (Gao et al., 2013; Gong et al., 2017), as gene flow may have frequently occurred among *Lilium* sections (Gong et al., 2017).

Nevertheless, our data imply that the incomplete lineage sorting caused by evolutionary radiation cannot be ruled out as a feasible cause for the polyphyly in the section-level taxonomic units within the genus *Lilium*. Briefly, both LTT and BAMM analyses showed that the acceleration of species divergence since the late Miocene (*ca*. 10.0 Ma) may have played an essential role in developing the rich species diversity of *Lilium*. Such radiative diversification most likely resulted in ancestral allelic polymorphisms being shared between closely related lineages or species (Rieseberg and Wendel, 1993; Soltis and Kuzoff, 1995; Soltis et al., 1996; Wendel and Doyle, 1998; Philippe et al., 2005), thereby resulting in phylogenetic incongruence and the non-monophyly of these *Lilium* sections.

This study also provides indirect evidence that convergent evolution is a non-negligible evolutionary trigger for the blurred taxonomic boundaries of these section-level taxonomic units defined by floral morphologies (Liu and Sun, 2019; Givnish et al., 2020). Specifically, S-DIVA analysis indicated that 10 cross-regional dispersals occurred during the evolution of *Lilium*. These dispersals likely created populations of genetically divergent species in new environmental conditions. Morphological convergence occurred in distinct *Lilium* taxa possessing highly similar morphological characteristics (Liu and Sun, 2019; Givnish et al., 2020), as adaptation to new habitats triggers colonizing populations to possess similar morphologies to already established populations (Givnish, 1997).

### 4.2 Biogeographic scenarios

Climate changes have profoundly impacted the evolution of organisms in various geological eras (Hooker et al., 2004; Allen et al., 2006; Linder, 2008; Svenning et al., 2015; Schluter and Pennell, 2017; Muellner-Riehl et al., 2019). Generally, large-scale climatic change can lead to geographic shifts in available habitats for plants (Donoghue et al., 2001; Ohlemüller, 2011; Nürk et al., 2015), thus triggering migration or local extinction, which in turn significantly affects the distribution of plant taxa and communities (Graham, 2011). Such biogeographic shifts are well evidenced in the evolution of *Lilium*, as our data suggest that the Neogene climatic changes played a crucial role in shaping the distribution of extant species in the genus.

Although previous studies based on nuclear ITS data suggested that the geographic origin of *Lilium* was in Southwest China and the QTP (Gao et al., 2013; Huang et al., 2018), our data revealed that the maternal most recent common ancestor of extant *Lilium* species might have been widely distributed throughout the temperate regions of the Northern Hemisphere in the middle Miocene (*ca.* 17.04 Ma), and has experienced multiple dispersal, extinction, and vicariance events over time. The crown node of *Lilium*, which was associated with a dispersal and an extinction event, was dated to the MMCO, the warmest interval of the last 23 Ma (Flower and Kennett, 1994; Zachos et al., 2001, 2008). Within the Northern Hemisphere, the global warming in the MMCO most likely led to the expansion of temperate forest zone toward high latitudes and elevations, as well as the northward expansion of tropical rainforests (Wang et al., 2021). Given that the winged seeds of *Lilium* are easily transported by wind over long distances, the substitution of vegetation zones along latitudinal and altitudinal gradients in the MMCO would trigger the migration to higher latitudes and elevations and local extinction of some ancestral populations encountering physical or ecological barriers to spread, and thus drove the early divergence of *Lilium*.

After MMCO, the global temperatures have gradually decreased since the Middle Miocene Climate Transition (MMCT, 15.97–11.61 Ma; Zachos et al., 2001, 2008). This climatic change resulted in the expansion of temperate biomes and aridification in the continental interior of the Northern Hemisphere (Herbert et al., 2016; Holbourn et al., 2018; Sun et al., 2020), which may have triggered the dispersal, vicariance, and extinction events within the earliest *Lilium* ancestral populations to diverge. This scenario is supported by molecular dating and S-DIVA analysis. The divergence of Clade I that formed the rudiment of the disjunction distribution between North American and Southwest China (including the QTP), resulting from a combination of a dispersal, a vicariance, and an extinction, occurred at 13.22 Ma; accompanied by a vicariance, the crown age of Clade II was dated at 12.73 Ma.

S-DIVA analysis identified five dispersal events within Clade I. Specifically, an intercontinental dispersal event from Southwest China and the QTP to North American was dated to 10.05 Ma. The remaining four dispersal events, which were respectively dated to 11.30 Ma, 10.47 Ma, 8.64 Ma, and 8.33 Ma, occurred within East Asia. All these dispersal events took place in the late Miocene, when the intensification of the Asian summer monsoon established a humid climate and caused a significant expansion of forests in East Asia (Sun and Wang, 2005; Wan et al., 2007; Jacques et al., 2011; 2015; Yao et al., 2011; Zhang et al., 2012). These climatic and environmental shifts likely created favorable habitats that facilitated the eastward and northward spread of *Lilium* from Southwest China to East, Central, South China and northern Indochina, as well as to North China and Northeast Asia. In the Miocene, the ancestor of *Lilium philadelphicum* was able migrate from East Asia to North America via the Bering land bridge (Tiffney, 1985; Gladenkov et al., 2002).

Within Clade II, the formation of the distribution range of extant species might also have been driven by ancient climatic changes described above. For instance, as the intensification of the Asian monsoon created a connection between forests from low to high latitudes of East Asia around the Oligocene–Miocene transition (Sun and Wang, 2005), the climatic cooling in the late Miocene would drive the southward and westward expansion of the ancestral populations of *Lilium davidii* to Central and Southwest China. Additionally, the global cooling in the Neogene also triggered the expansion of temperate forests in the Northern Hemisphere, which built a connection of temperate forests between Europe and East Asia in the early Pliocene (Wan et al., 2007). The availability of this ecological corridor may have contributed to the migration of the ancestor of *Lilium bulbiferum* to Europe.

### 4.3 Historical events responsible for the formation of uneven distribution in *Lilium*

Following the MMCT, global temperatures have continuously decreased since the late Miocene (Flower and Kennett, 1994; Pearson and Palmer, 2000; Zachos et al., 2001, 2008; Lewis et al., 2007; You et al., 2009; Herbert et al., 2016; Holbourn et al., 2018). This climate change is assumed to have led to the expansion of temperate habitats and proliferation of temperate biomes; as a result, the broader habitats may have triggered rapid species diversification in the temperate regions (Sun et al., 2020). Given the lack of empirical studies, more evidence is needed to confirm whether the global climate cooling since the late Miocene has generally contributed to the diversification of plant taxa adapted to temperate climates in the Northern Hemisphere (Sun et al., 2020).

Both LTT and BAMM analyses showed that the species diversification rate of *Lilium* has accelerated since approximately 10.0 Ma, around the late Miocene. This shift in species diversification rate appears in parallel with the global cooling that followed the MMCT (Flower and Kennett, 1994; Pearson and Palmer, 2000; Zachos et al., 2001, 2008; Lewis et al., 2007; You et al., 2009), the intensification of monsoonal climate in East Asia (Sun and Wang, 2005; Wan et al., 2007; Jacques et al., 2011; Yao et al., 2011; Zhang et al., 2012; Lu and Guo, 2013; Wang et al., 2017), and the further uplift of the QTP (Harrison et al., 1992; An et al., 2001). Thus, the acceleration of species diversification rate observed in *Lilium* may have been jointly triggered by these climatic and geological events.

Globally, both ecological and climatic heterogeneity are the basis for forming species diversity (Schluter, 2009, 2016; Goldberg et al., 2011). From this perspective, the global cooling since the late Miocene and the resultant expansion of temperate biomes may have provided more available habitats for species diversification in *Lilium*. At the same time, global cooling led to regional aridification of inlands of the Northern Hemisphere (Schluter, 2009, 2016), which likely fragmented habitats of *Lilium* and promoted vicariance to escalate speciation.

Regionally, the QTP rose from the late Miocene to the early Pliocene, which further strengthened the monsoonal climate in East Asia (Harrison et al., 1992; An et al., 2001; Spicer, 2017; Spicer et al., 2020, 2021). The uplift of QTP created heterogenous habitats in East Asia, particularly in Southwest China and the QTP (Hong and Blackmore, 2015; Wen et al., 2014), and the intensification of the summer monsoon established favorable humid climate over much of East Asia (Sun and Wang, 2005; Wan et al., 2007; Jacques et al., 2011; Yao et al., 2011; Zhang et al., 2012). Such complex geological, ecological, and environmental diversity in East Asia is proposed to have driven rapid diversification of a wide spectrum of plant taxa (Sun and Wang, 2005; Wen et al., 2014; Favre et al., 2015; Xing and Ree, 2017; Ji et al., 2019, 2021, 2023; Li et al., 2021), and facilitated species radiation in *Lilium*. At the same time, the uplift of QTP simultaneously led to arid environment in Asia inland (Sun and Wang, 2005; Zheng and Yao, 2005; Li, 2006; Liu and Dong, 2013), and thus would cause local reduction of *Lilium* species in central Asia. As a result, *Lilium* species diversity is higher in East Asia than in Central Asia, Europe, and North America. The inferred biogeographic scenarios and evolutionary diversification of *Lilium* provide new case study to justify the importance of the Neogene uplift of QTP and its induced climatic changes in the formation of uneven distribution of plant diversity in the Northern Hemisphere.

## 5 Conclusions

A robust plastome backbone phylogeny of *Lilium* was reconstructed in this study, providing new insights into classification, historical biogeography, and evolutionary diversification of the genus. Our findings imply that the evolution of *Lilium* experienced incomplete lineage sorting and morphological convergence. The co-occurrence of these evolutionarily complicated events may be responsible for the non-monophyly and blurred taxonomic boundaries of currently recognized section-level taxonomic units within *Lilium*. Additionally, our results suggest that the Neogene climatic and geological events in East Asia played important roles in shaping the distribution of *Lilium* and in triggering its evolutionary radiation. These events include the successive uplift of the QTP and strengthening of the monsoon climate in East Asia. Our findings represent a case study for understanding how unique geological and climatic events have created uneven distributions of plant diversity in the Northern Hemisphere. Nevertheless, our use of plastome data and incomplete sampling of *Lilium* has left several historical problems in the phylogeny of *Lilium* unresolved. To better understand the evolution and classification of *Lilium*, a taxonomic sampling with greater coverage of *Lilium* species and the use of nuclear genome-scale data are needed to robustly recover the phylogeny and to critically explore the evolutionary complexity of the genus.

## Authors’ contributions

Y. Ji conceived the study; N. Zhou and K. Miao collected and analyzed the data; N. Zhou, K. Miao, and Y. Ji wrote the manuscript; C. Liu, L. Jia, J. Hu. Y. Huang, and Y. Ji discussed the results and revised the manuscript. All authors have read and approved the manuscript.

## Data availability

The sequences generated in this study are available at NCBI database and the accession numbers are presented in Table S1 and Table S2.

## Declaration of competing interest

The authors declare no conflict of interest.

## Figures and Tables

**figs1:**
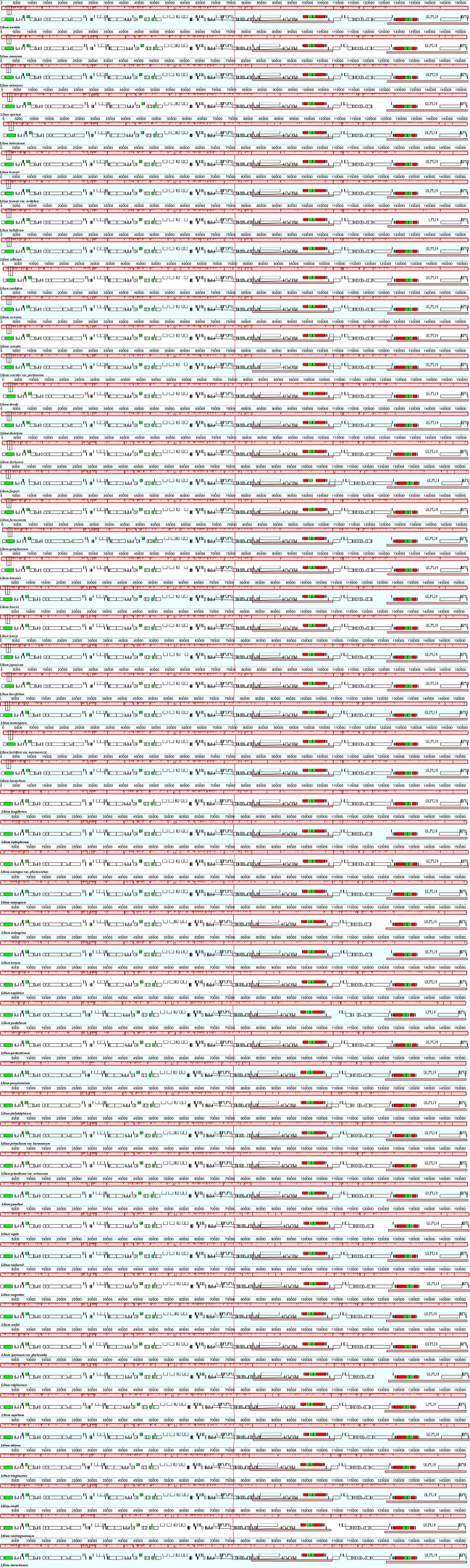


**figs2:**