# Supplementary material for: Historical biogeography and evolutionary diversification of Lilium (Liliaceae): New insights from plastome phylogenomics
Source: Plant Divers. 2023 Aug 3;46(2):219–28. doi: 10.1016/j.pld.2023.07.009 (PMC11128834; doi:10.1016/j.pld.2023.07.009)
Supplement: Multimedia component 1 [file mmc1.docx]

**Table S1**. Samples newly sequenced in the study, with voucher and source information, and GenBank accessions.

| Species | GenBank Accession | Voucher | Locality |
| --- | --- | --- | --- |
| *Lilium amabile* | OP784235 | AHC2019016 | Panshi, Jilin |
| *Lilium brownii* | OP784240 | Ji et Wang 9 | Tengchong, Yunnan |
| *Lilium callosum* | OP784236 | AHC2019017 | Panshi, Jilin |
| *Lilium cernuum* | OP784238 | AHC2019020 | Panshi, Jilin |
| *Lilium concolor*var.*partheneion* | OP784245 | Caow5450 | DanDong, Liaoning |
| *Lilium distichum* | OP784239 | AHC2019021 | Panshi, Jilin |
| *Lilium lancifolium* | OP784241 | 2020078 | Kunming, Yunnan |
| *Lilium lankongense* | OP784243 | 2020092 | Diqing Tibetan Autonomous Prefecture, Yunnan |
| *Lilium leichtlinii* var. *maximowiczii* | OP756529 | AHC2019012 | Panshi, Jilin |
| *Lilium leucanthum* | OP784242 | 2020091 | Shiyan, Hubei |
| *Lilium pensylvanicum* | OP784234 | AHC2019014 | Panshi, Jilin |
| *Lilium pumilum* | OP784237 | AHC2019019 | Panshi, Jilin |
| *Lilium speciosum* var. *gloriosoides* | OP784246 | TanCM3240 | Jiujiang, Jiangxi |
| *Lilium wardii* | OP784244 | ZhangDC-07ZX-1738 | Linzhi, Xizang |
