# Supplementary material for: Historical biogeography and evolutionary diversification of Lilium (Liliaceae): New insights from plastome phylogenomics
Source: Plant Divers. 2023 Aug 3;46(2):219–28. doi: 10.1016/j.pld.2023.07.009 (PMC11128834; doi:10.1016/j.pld.2023.07.009)
Supplement: Multimedia component 2 [file mmc2.docx]

**Table S2.** Publicly available plastome sequences download from NCBI GenBank database and National Genomics Data Center.

| Species | Accession No. | Species | Accession No. |
| --- | --- | --- | --- |
| ***Lilium*** | | | |
| *Lilium amoenum* | MT880912 | *Lilium formosanum* | MT261162 |
| *Lilium anhuiense* | MW890005 | *Lilium gongshanense* | MK493297 |
| *Lilium apertum* | MK493293 | *Lilium hansonii* | MT261163 |
| *Lilium bakerianum* | KY748301 | *Lilium henrici* | MH136807 |
| *Lilium brownii* var*. viridulum* | MN906759 | *Lilium henryi* | KY748302 |
| *Lilium bulbiferum* | MW465412 | *Lilium humboldtii* | GWHBGXT01000000 |
| *Lilium canadense* | GWHBGXX01000000 | *Lilium japonicum* | MT261164 |
| *Lilium candidum* | MK753244 | *Lilium longiflorum* | MW900178 |
| *Lilium ciliatum* | GWHBGXW01000000 | *Lilium lophophorum* | MK493298 |
| *Lilium concolor* | MZ676707 | *Lilium maculatum var. maculatum* | GWHBGXE01000000 |
| *Lilium davidii* | MW890008 | *Lilium martagon* | GWHBGXN01000000 |
| *Lilium davidii var. willmottiae* | GWHBGZL01000000 | *Lilium martagon* var. *pilosiusculum* | MF964219 |
| *Lilium duchartrei* | KY748300 | *Lilium matangense* | MN745201 |
| *Lilium fargesii* | KX592156 | *Lilium meleagrina* | MK493299 |
| *Lilium monadelphum* | GWHBGXM01000000 | *Lilium souliei* | MW007720 |
| *Lilium nanum* | MK493300 | *Lilium speciosum* | GWHBGXI01000000 |
| *Lilium nepalense* | MK493301 | *Lilium speciosum var. gloriosoides* | GWHBGXU01000000 |
| *Lilium pardalinum* | MH029495 | *Lilium stewartianum* | MN745202 |
| *Lilium pardanthinum* | MG704135 | *Lilium sulphureum* | MK493304 |
| *Lilium parryi* | GWHBGXK01000000 | *Lilium superbum* | KP462883 |
| *Lilium philadelphicum* | KY940847 | *Lilium szovitsianum* | GWHBGXG01000000 |
| *Lilium primulinum* var. *burmanicum* | MZ188968 | *Lilium taliense* | KY009938 |
| *Lilium primulinum* var. *ochraceum* | KY748298 | *Lilium tsingtauense* | KU230438 |
| *Lilium regale* | MK493302 | *Lilium ukeyuri* | GWHBGYA01000000 |
| *Lilium rosthornii* | MW890009 | *Lilium washingtonianum* | MG590100 |
| *Lilium sargentiae* | MK493303 |  |  |
| **Outgroup** |  |  |  |
| *Cardiocrinum cathayanum* | KX575836 | *Fritillaria persica* | NC_037039 |
| *Cardiocrinum cordatum* | NC_033898 | *Fritillaria yuzhongensis* | MK258139 |
| *Cardiocrinum giganteum* | NC_033896 | *Notholirion bulbuliferum* | MW890007 |
| *Fritillaria davidii* | MK258145 | *Notholirion macrophyllum* | MH011354 |
| *Fritillaria eduardii* | NC_037038 | *Notholirion thomsonianum* | MZ128661 |
| *Fritillaria karelinii* | KX354691 | *Tulipa altaica* | MW077741 |
| *Fritillaria maximowiczii* | MK258138 | *Tulipa iliensis* | MW077740 |
| *Fritillaria meleagroides* | NC_037040 | *Lloydia tibetica* | MW890011 |
