# Supplementary material for: Historical biogeography and evolutionary diversification of Lilium (Liliaceae): New insights from plastome phylogenomics
Source: Plant Divers. 2023 Aug 3;46(2):219–28. doi: 10.1016/j.pld.2023.07.009 (PMC11128834; doi:10.1016/j.pld.2023.07.009)
Supplement: Multimedia component 3 [file mmc3.docx]

**Table S3.** Taxonomic position and distribution information of the genus *Lilium*.

| Species | Section | Natural distribution^1^ | Area^2^ |
| --- | --- | --- | --- |
| *Lilium amabile* | *Sinomartagon* | SE. Liaoning; Korea | C |
| *Lilium amoenum* | *Sinomartagon* | Yunnan | A |
| *Lilium anhuiense* | *Leucolirion* | S. Anhui | B |
| *Lilium apertum* | *Nomocharis* | SW. Sichuan, Xizang, NW. Yunnan; N. Myanmar | A |
| *Lilium bakerianum* | *Sinomartagon* | Guizhou, Sichuan, Yunnan; Myanmar | A |
| *Lilium brownii* | *Archelirion* | Anhui, Fujian, Gansu, Guangdong, Guangxi, Guizhou, Hebei, Henan, Hubei, Hunan, Jiangsu, Jiangxi, Shaanxi, Shanxi, Sichuan, Yunnan, Zhejiang | ABC |
| *Lilium brownii* var*. viridulum* | *Archelirion* | Anhui, Fujian, Gansu, Guangxi, Guizhou, Hebei, Henan, Hubei, Hunan, Jiangsu, Jiangxi, Shaanxi, Shanxi, Sichuan, Yunnan, Zhejiang | B |
| *Lilium bulbiferum* | *Liriotypus* | Central Europe to S. Italy | D |
| *Lilium callosum* | *Sinomartagon* | Anhui, Guangdong, Guangxi, Henan, Jiangsu, Jilin, Liaoning, Nei Mongol, Taiwan, Zhejiang; Japan, Korea, Russia | BC |
| *Lilium canadense* | *Pseudolirium* | E. Canada to E. U.S.A. | E |
| *Lilium candidum* | *Liriotypus* | S. North Macedonia to SW. Turkey, Lebanon to Israel | D |
| *Lilium cernuum* | *Sinomartagon* | Jilin, Liaoning; Korea, Russia | C |
| *Lilium ciliatum* | *Liriotypus* | NE. Turkey | D |
| *Lilium concolor* | *Sinomartagon* | Hebei, Heilongjiang, Henan, Hubei, Jilin, Liaoning, Nei Mongol, Shaanxi, Shandong, Shanxi, Yunnan; Japan, Korea, Mongolia, Russia | ABC |
| *Lilium concolor*var.*partheneion* | *Sinomartagon* | S. Siberia to N. China and Japan | C |
| *Lilium davidii* | *Sinomartagon* | Gansu, Guizhou, Henan, W. Hubei, S. Shaanxi, Shanxi, Sichuan, Yunnan | ABC |
| *Lilium davidii var. willmottiae* | *Sinomartagon* | W Hubei, S Shaanxi, E Sichuan, Yunnan. | AB |
| *Lilium distichum* | *Martagon* | Heilongjiang, Jilin, Liaoning; Korea, Russia | C |
| *Lilium duchartrei* | *Sinomartagon* | Gansu, Hubei, S Shaanxi, Sichuan | AB |
| *Lilium fargesii* | *Sinomartagon* | Hubei, Shaanxi, Sichuan, Yunnan | B |
| *Lilium formosanum* | *Leucolirion* | Taiwan | B |
| *Lilium gongshanense* | *Nomocharis* | Yunnan | A |
| *Lilium hansonii* | *Martagon* | S. Jilin; Korea | C |
| *Lilium henrici* | *Sinomartagon* | W. Sichuan, NW. Yunnan | A |
| *Lilium henryi* | *Sinomartagon* | Guizhou, Hubei, Jiangx | AB |
| *Lilium humboldtii* | *Pseudolirium* | California to Mexico (N. Baja California) | E |
| *Lilium japonicum* | *Archelirion* | Central and S. Japan | C |
| *Lilium lancifolium* | *Sinomartagon* | Anhui, Gansu, Guangxi, Hebei, Henan, Hubei, Hunan, Jiangsu, Jiangxi, Jilin, Qinghai, Shaanxi, Shandong, Shanxi, Sichuan, Xizang, Zhejiang; Japan, Korea | ABC |
| *Lilium lankongense* | *Sinomartagon* | SE. Xizang, NW. Yunnan | A |
| *Lilium leichtlinii* var. *maximowiczii* | *Sinomartagon* | Hebei, Jilin, Liaoning, Shaanxi; Japan, Korea, Russia | C |
| *Lilium leucanthum* | *Leucolirion* | Gansu, Hubei, Sichuan | B |
| *Lilium longiflorum* | *Leucolirion* | Japan (Yakushima, Tanegashima) to Philippines (Batan Islands, N. Luzon) | B |
| *Lilium lophophorum* | *Sinomartagon* | Sichuan, Xizang, Yunnan | A |
| *Lilium maculatum var. maculatum* | *Sinomartagon* | Japan (Honshu) | C |
| *Lilium martagon* | *Martagon* | SW. & Central Europe to Mongolia | D |
| *Lilium martagon* var. *pilosiusculum* | *Martagon* | N. Xinjiang; Mongolia, Russia | C |
| *Lilium matangense* | *Sinomartagon* | NW. Sichuan | A |
| *Lilium meleagrina* | *Nomocharis* | SE. Tibet to China (Sichuan, NW. Yunnan) | A |
| *Lilium monadelphum* | *Liriotypus* | Krym, Caucasus. | D |
| *Lilium nanum* | *Sinomartagon* | Sichuan, Xizang, Yunnan; Bhutan, Myanmar, Nepal, Sikkim | A |
| *Lilium nepalense* | *Sinomartagon* | S. Xizang, SE. and W. Yunnan; Bhutan, N. India, Myanmar, Nepal, Sikkim | A |
| *Lilium pardalinum* | *Pseudolirium* | Oregon to Mexico (N. Baja California) | E |
| *Lilium pardanthinum* | *Nomocharis* | SW. Sichuan, NW. Yunnan | A |
| *Lilium parryi* | *Pseudolirium* | S. California, SE. Arizona, Mexico (Baja California Norte, Sonora) | E |
| *Lilium pensylvanicum* | *Daurolirion* | Hebei, Heilongjiang, Jilin, Liaoning, Nei Mongol; Japan, Korea, Mongolia, Russia | C |
| *Lilium philadelphicum* | *Pseudolirium* | Canada to W. Central & E. USA | E |
| *Lilium primulinum* var. *burmanicum* | *Sinomartagon* | Yunnan; Myanmar, Thailand | A |
| *Lilium primulinum* var. *ochraceum* | *Sinomartagon* | Guizhou, Sichuan, NW. Yunnan | A |
| *Lilium pumilum* | *Sinomartagon* | Gansu, Hebei, Heilongjiang, Henan, Jilin, Liaoning, Nei Mongol, Ningxia, Qinghai, Shaanxi, Shandong, Shanxi; Korea, Mongolia, Russia | BC |
| *Lilium regale* | *Leucolirion* | Sichuan | A |
| *Lilium rosthornii* | *Sinomartagon* | Guizhou, Hubei, Sichuan | B |
| *Lilium sargentiae* | *Leucolirion* | Sichuan, SE Xizang, Yunnan | A |
| *Lilium souliei* | *Sinomartagon* | Sichuan, SE Xizang, Yunnan | A |
| *Lilium speciosum* | *Archelirion* | SE. China, Taiwan, Japan (Shikoku, Kyushu) | BC |
| *Lilium speciosum* var*. gloriosoides* | *Archelirion* | SE. China, Taiwan; Japan | B |
| *Lilium stewartianum* | *Sinomartagon* | NW. Yunnan | A |
| *Lilium sulphureum* | *Leucolirion* | Guangxi, Guizhou, Sichuan, Yunnan; Myanmar | AB |
| *Lilium superbum* | *Pseudolirium* | E. Central & E. USA | E |
| *Lilium szovitsianum* | *Liriotypus* | NE. Türkiye to Caucasus | D |
| *Lilium taliense* | *Sinomartagon* | Sichuan, Xizang, Yunnan | A |
| *Lilium tsingtauense* | *Martagon* | Anhui, Shandong; Korea | BC |
| *Lilium ukeyuri* | *Archelirion* | N. Nansei-shoto | C |
| *Lilium wardii* | *Sinomartagon* | Guizhou, Sichuan, SE. Xizang | A |
| *Lilium washingtonianum* | *Pseudolirium* | W. Oregon to N. Central California | E |

1 The natural distributions of the taxa were obtained from the Flora of China (2000) and POWO (https://powo.science.kew.org/).

2 A, Southwest China and Himalayas; B, East, Central, South China and northern Indochina; C, North China and Northeast Asia; D, Central Asia and Europe; E,North America.
